# Radial Head Incarceration After Closed Reduction of a Pediatric Elbow Dislocation With a Radial Neck Fracture: A Case Report

**DOI:** 10.5435/JAAOSGlobal-D-21-00319

**Published:** 2022-07-12

**Authors:** Denver B. Kraft, Evan D. Sheppard

**Affiliations:** From the Department of Orthopaedic Surgery, MedStar Georgetown University Hospital; Washington, DC (Dr. Kraft), and the Department of Pediatric Orthopaedics, Pediatric Specialists of Virginia, Inova Fairfax Hospital, Fairfax, VA (Dr. Sheppard).

## Abstract

**Case:**

A 10-year-old girl sustained a radial neck fracture with a posterior elbow dislocation. She was treated with closed reduction of the elbow with subsequent intra-articular displacement of the radial head, which necessitated open reduction and pinning of the radial neck fracture.

**Conclusion:**

Displacement of the radial neck fracture from impingement of the capitellum on the anterior radial head during closed reduction of the elbow dislocation is a rare injury pattern. It is important to examine the radial neck in high-energy posterior elbow dislocations before attempted reduction. We present a case with imaging depicting the injury mechanism and successful management with subsequent open reduction and fixation of the radial neck fracture.

Radial neck fractures represent 1% of all pediatric fractures and 5% to 10% of elbow injuries in children.^1-3^ Injury typically occurs after a fall from height onto an outstretched arm with a possible torsional component. Initial treatment involves closed reduction with progression to percutaneous or open reduction if adequate results cannot be achieved through closed means.^4^ There is debate as to which injury characteristics may preclude successful closed management and should proceed directly to open reduction to minimize articular damage.^5-7^ Increased displacement increases the risk for postoperative complications, such as osteonecrosis,^8^ nonunion, overgrowth, physeal arrest,^9^ and stiffness,^1,5,6^ and up to half of children with radial neck fractures have subsequent limited forearm rotation.^10,11^ Risk for stiffness and loss of motion is due to trauma at the time of injury but can be exacerbated by subsequent trauma from attempted closed reductions.^4^ It is generally accepted that residual angulation over 30° or translation over 2 mm necessitates open reduction.^4^ In a retrospective study on pediatric radial neck fractures by Zimmerman et al, the authors found that initial displacement and age older than 10 years were associated with poor outcomes while another study by Gutierrez found only initial displacement as a predictor of poor outcome.^5,7^

We present a pediatric posterior elbow dislocation with Salter-Harris type II radial neck fracture complicated by post–elbow reduction radial head incarceration within the ulnohumeral joint. The closed reduction fluoroscopy demonstrates the mechanism of the rare Jeffery type II lesion in which the capitellum impinges on the anterior lip on the radial head during reduction of a posterior dislocation, causing a 90° backward tilt of the head.^12,13^ After transfer to our facility, the decision was made to proceed urgently to open reduction with pinning to minimize additional articular damage and because of the difficulties associated with closed management of Jeffery type II lesions.

## Case Report

**Figure 1:**
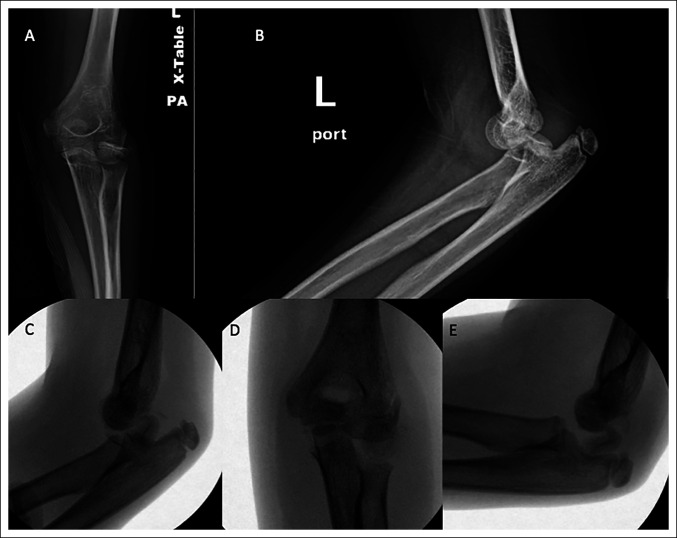
Images presenting left elbow injury radiographs. Radiographs showing anteroposterior (**A**) and lateral (**B**) views of the left elbow depicting a posterior elbow dislocation and a Salter-Harris II radial neck fracture.

**Figure 2:**
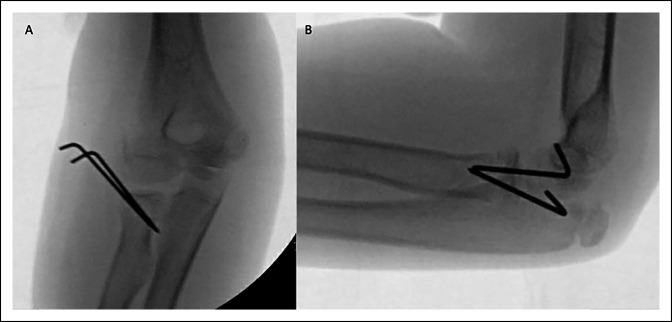
Fluoroscopic imaging of the closed reduction of the elbow dislocation with displacement of the radial neck fracture. Mid-reduction lateral image of the elbow showing displacement of the radial neck fracture (**A**). Postreduction anteroposterior (**B**) and lateral (**C**) views of the elbow showing a displaced and incarcerated radial head.

A 10-year-old girl presented to an outside emergency department with left elbow pain after falling off of a horse. She was neurovascularly intact and found to have a posterior elbow dislocation with a Salter-Harris II fracture of the radial neck (Figure 1). Reduction attempt of the posterior elbow dislocation displaced the radial neck fracture, with the capitellum remaining interposed between the radial shaft and head (Figure 2). The patient was transferred to our hospital, and the decision was made to urgently proceed to open reduction to decrease the risk of osteonecrosis to the radial head and to decrease additional chondral damage.

With the forearm pronated, a lateral incision was used to dissect down to the interval between anconeus and extensor carpi ulnaris. The capsule was already disrupted, and the elbow was dislocated. A blunt instrument was used to locate the proximal radius, and traction was used to reduce the radius toward the capitellum. The radial head was encountered resting at 90° to the axis of the radial shaft. Continued traction with digital guidance and a dental pick helped to align the radial head back to the shaft; the correct rotation was achieved by aligning the metaphyseal spike of the Salter-Harris II fracture. There was a small cuff of tissue attached to the medial aspect of the radial head, which was preserved. A Kirschner wire was then inserted, anterograde, through the skin, outside of the incision, from the periphery of the radial head articular cartilage toward the metadiaphysis for fixation. The elbow was taken through a gentle range of motion, to assess the stability of the elbow joint, and the fracture was noted to lose its anatomic alignment when the elbow was extended, and so an additional Kirschner wire was placed to increase stability. The Kirschner wires were placed in the nonarticulating zone of the radial head as determined by the 90° arc from the radial styloid to the Lister tubercle distally^4^ (Figure 2). The lateral ligaments were repaired, and the wound was closed in a standard fashion with the pins bent outside the body. The elbow was taken through a range of motion; was stable in flexion, extension, pronation, and supination; and was placed in a well-padded posterior splint with side struts.

**Figure 3:**
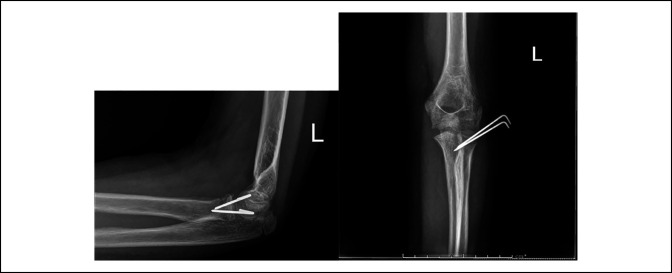
Images showing immediate postoperative fluoroscopic imaging of the elbow. Radiographs showing anteroposterior (**A**) and lateral (**B**) views of the left elbow after open reduction and pinning of the radial neck fracture.

**Figure 4:**
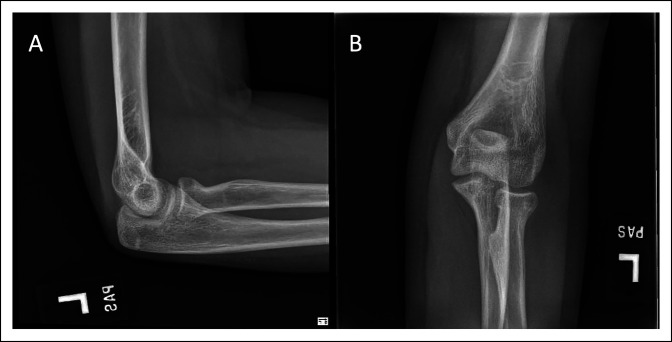
Images showing follow-up radiographs 3 months after fixation. Anteroposterior (**A**) and lateral (**B**) views of the left elbow after removal of the pins, with interval healing of the radial neck fracture and excellent radial neck, ulnohumeral, and radiodcapitellar alignment with no signs of displacement or osteonecrosis.

The patient was followed up at 1 week, 4 weeks, 6 weeks, 3 months, and 6 months. At 1 week, radiographs showed stable alignment of the fracture and no implant complication and the splint was overwrapped with fiberglass. At 4 weeks, periosteal reaction was seen over the proximal radius, her pins were pulled, and she was placed back in a long arm cast (Figure 3). At 6 weeks, she was neurovascularly intact, the incision was well healed, and she was transitioned to a hinged elbow brace with 30° range of motion; on imaging, the radial neck fracture was well aligned with callous formation evident. At 3 months, she had full flexion and pronation. She was lacking 5° extension and supination. She was pain-free and stable to varus and valgus stress. Imaging at her 3-month postoperative visit revealed a well-healed radial neck with excellent radial neck, ulnohumeral, and radiocapitellar alignment with no signs of displacement or osteonecrosis (Figure 4). This was maintained at her 6-month evaluation.

## Discussion

Open reduction of severely displaced radial neck fractures is encouraged by some authors to avoid injury because of repetitive closed manipulation.^14^ However, some studies document a correlation between open reduction and poor functional outcome. Whether poor results are a consequence of the treatment or the magnitude of initial injury is unclear, and no study has documented open reduction being independently associated with poor outcomes.^1,5-7,15^ Surgical treatment options include percutaneous pin reduction^16,17^; elastic intramedullary nailing^18^; and open reduction with or without suture, plate, screw, or Kirschner wire fixation.^3,5,6,19^ In the presented case, owing to the concomitant elbow dislocation and incarceration of the radial head in the joint, an open reduction and pinning was the chosen method of treatment.

The literature surrounding the extensive variation in fracture pattern and specific complications associated with each type of radial neck fracture with or with associated elbow injuries is sparse. Our case is consistent with a multicenter retrospective study by Fitoussi et al,^20^ which found that radial neck fractures after horse-riding accidents in the pediatric population were more severe, more often require open reduction, and are associated with an increased incidence of osteoarticular lesions including dislocation than those caused by other mechanisms. Jeffery type 2 radial neck injuries are those that occur during the reduction of a posterior elbow dislocation and occur in only 2% to 4% of all radial neck fractures, and in 80% of these cases, there is no evidence of dislocation despite the probable mechanism.^21^ This is a reported case of the rare Jeffery type 2 lesion in which there is documentation of the posterior dislocation and fluoroscopic imaging highlighting the mechanism of radial neck fracture displacement during attempted reduction. Attempted closed reduction of the Jeffery type 2 radial neck fracture has led to complete radial head inversion in many instances.^14,21^ In this case, the patient was treated with open reduction and antegrade Kirschner wire fixation and had good results at the 6-month follow-up with fracture healing, no evidence of osteonecrosis, and good postoperative range of motion.

In conclusion, this case provides insight into the complications that can arise after attempted closed reduction of an elbow dislocation associated with radial neck fracture and the subsequent management of the displaced radial head. Multiple attempts at closed reduction may further traumatize the elbow and increase the risk of postoperative complications, especially with an increasing degree of displacement; therefore, open reduction and pinning provide an appealing alternative treatment with good postoperative functional outcome in this presented case. It is important to closely scrutinize the radial neck in posterior elbow dislocations before reduction. It may be prudent to reduce elbow dislocations with radial neck fractures in the operating room to address radial head displacement and entrapment urgently, if this complication arises.
